# Constructing Large 2D Lattices Out of DNA-Tiles

**DOI:** 10.3390/molecules26061502

**Published:** 2021-03-10

**Authors:** Johannes M. Parikka, Karolina Sokołowska, Nemanja Markešević, J. Jussi Toppari

**Affiliations:** Nanoscience Center, Department of Physics, University of Jyväskylä, P.O. Box 35, 40014 Jyväskylä, Finland; johannes.m.parikka@jyu.fi (J.M.P.); karolina.x.sokolowska@jyu.fi (K.S.); nemanja.n.markesevic@jyu.fi (N.M.)

**Keywords:** DNA self-assembly, DNA origami, DNA nanotechnology, lattice, hierarchy, complexity, lithography

## Abstract

The predictable nature of deoxyribonucleic acid (DNA) interactions enables assembly of DNA into almost any arbitrary shape with programmable features of nanometer precision. The recent progress of DNA nanotechnology has allowed production of an even wider gamut of possible shapes with high-yield and error-free assembly processes. Most of these structures are, however, limited in size to a nanometer scale. To overcome this limitation, a plethora of studies has been carried out to form larger structures using DNA assemblies as building blocks or tiles. Therefore, DNA tiles have become one of the most widely used building blocks for engineering large, intricate structures with nanometer precision. To create even larger assemblies with highly organized patterns, scientists have developed a variety of structural design principles and assembly methods. This review first summarizes currently available DNA tile toolboxes and the basic principles of lattice formation and hierarchical self-assembly using DNA tiles. Special emphasis is given to the forces involved in the assembly process in liquid-liquid and at solid-liquid interfaces, and how to master them to reach the optimum balance between the involved interactions for successful self-assembly. In addition, we focus on the recent approaches that have shown great potential for the controlled immobilization and positioning of DNA nanostructures on different surfaces. The ability to position DNA objects in a controllable manner on technologically relevant surfaces is one step forward towards the integration of DNA-based materials into nanoelectronic and sensor devices.

## 1. Introduction

One of the most inspiring phenomena of all living organisms is that of molecular self-assembly. It is fascinating how Nature’s highly sophisticated molecular design enables the formation of self-organized, hierarchical structures out of a limited variety of components [1,2]. In general, a precise arrangement and organization of components forming a biological assembly is the key to its functionality [2]. For example, chains of DNA form a double helix that encodes our genome, which is used to fabricate amino acid chains that further fold into complex shapes to form functional proteins, and finally, these and all other biomolecules are comprised into cells, creating eventually plants, animals, and humans.

In Nature, the principles of self-assembly are generally governed by weak and noncovalent interactions such as hydrogen bonding, hydrophobic interactions, and van der Waals interactions. The aforementioned set of interactions is vital since it enables an easy breakage and reformation of bonds at around room temperature, thus allowing a system to relax into a minimum energy state sometimes even through the most complex routes. This makes it possible to form highly sophisticated structures through trial and error. Understanding how these weak molecular interactions guide the assembly process has opened a new window of opportunities to create novel design strategies towards devices with desired functions.

Inspired by the self-assembly found in nature, scientists have used a variety of molecules as building blocks to form higher-ordered, more sophisticated, designed constructions [3,4]. Among these molecules, DNA remains the most attractive candidate for nanoscale constructions due to its resolved sub-nanometer structural details and fairly comprehensive understanding of the involved interaction forces, allowing precise engineering [5,6]. A single-stranded DNA (ssDNA) is a sequence of linked monomers, nucleotides. When linked, they a sugar-phosphate backbone with one of the four possible organic bases, adenine (A), cytosine (C), thymine (T), or guanine (G), attached to each sugar ring. While all the bonds between the nucleotides in a single DNA strand are covalent and thus very stable within the interplay of self-assembly, the formation of distinguished double helices, governed by selective but easily detachable hydrogen bonds, leads to the essential reversible assembly. The nature of these hydrogen bonds also makes the DNA self-assembly predictable and specific, and consequently easy to program. Moreover, the structure of DNA combines simultaneously stiffness and flexibility, making it an ideal building block that ensures structural stability but still tolerates small strains within the construction [7]. Besides, the fast and cost-effective synthesis of oligonucleotides allowed the rapid development of structural DNA nanotechnology and the design of a variety of DNA nanostructures with a nanoscale precision [8].

**Figure 1:**
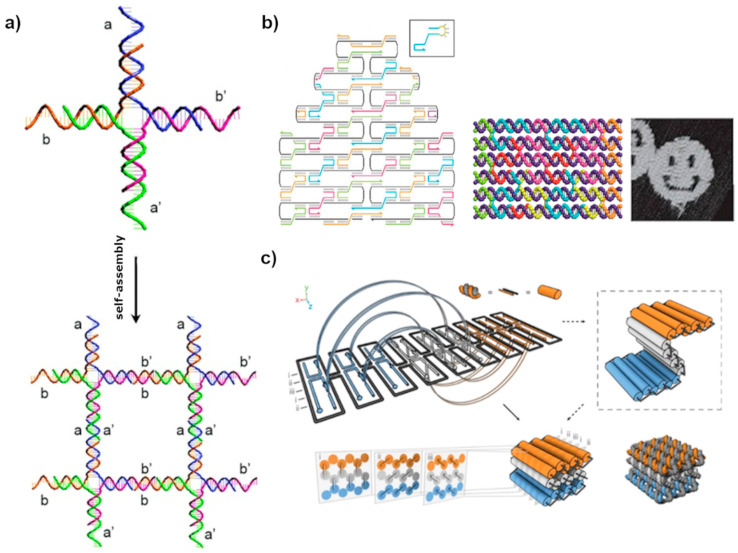
(**a**) Fundamental structural motif of immobile branched DNA junction combined with sticky ends to self-assemble into 2D lattices [9,38]. (**b**) DNA origami assembly method. An example of a DNA origami scaffold (black line) and staples (colored line) strand routing involving several crossover points where the scaffold or a staple strand crosses from one helix to another thus connecting them together. Adapted by permission from Springer: Nature [11], copyright 2006. Crossovers are topologically similar to the branched junction and hold the origami on the desired shape. The scaffold and the staples are mixed together resulting in the self-assembled structure as shown in the 3D schematic view. Adapted with permission from [39]. Copyright 2017 American Chemical Society. Atomic force microscope (AFM) image of one of the assembled DNA origami structures. Adapted with permission from [11]. Copyright 2006 Springer: Nature. (**c**) Schematics of the design of a three-dimensional origami structure. Adapted with permission from [12] copyright 2006 Springer: Nature.

Seeman et al. [9] introduced the first concept of DNA nanotechnology in the 1980s by constructing geometric objects and periodic 2D or 3D lattices based on multi-arm motifs (Figure 1a). The idea comprised of single-stranded overhangs, or “sticky ends”, attached at the ends of the branched DNA junctions serving as a connective glue to assemble the structure. Since then, the field has flourished rapidly with new designs and concepts to construct DNA-based systems [10]. A significant impact on the complexity of structural DNA was introduced by an invention of the DNA origami technique by Rothemund in 2006 (Figure 1b) [11]. He reported a method where several kilobases long ssDNA, known as a scaffold, was folded into different designed 2D shapes with help of hundreds of synthetic oligonucleotides referred to as staple strands. This accomplishment truly changed the scenery of structural DNA nanotechnology bringing new possibilities for forming complex structures, especially after Shih’s research group extended the origami technique into 3D structures as shown in Figure 1c [12]. Since then DNA origami has proven its versatility as a building material in a nanometer-scale world by engineering custom structures with control even at a single-molecule level [5,13,14,15]. DNA origami nanostructures have been applied in variety of applications, such as weak and strong light-matter coupling research [16,17], chiral objects formation [18,19], reconfigurable plasmonic devices [20], drug delivery [21,22], Förster energy transfer research [23,24] and bio-applications [25,26].

Since the invention of the very first self-assembled DNA nanostructures, the aim has been to achieve larger assemblies made up of individual DNA units. Even though the origami structures are already about 100 nm in size what makes them much bigger than the earlier DNA motifs of the size of ~10 nm or plain dsDNA with a thickness of ~2 nm, the size of an individual origami structure is still limited by the length of the scaffold. To scale up the structures up to micrometers, many attempts have been reported, including extending the scaffold length with a variety of biotechnological methods, such as rolling circle amplification (RCA) or polymerase chain reaction (PCR) [27,28,29]. Nevertheless, these structures are still limited in size. An alternative method to overcome this problem involves hierarchical self-assembly [30,31,32,33,34,35,36,37], where the individual self-assembled DNA origami structures work as the construction units or tiles to form a larger organized structure through higher-order self-assembly strategies.

The driving forces contributing to the above-mentioned higher-order self-assembly are partially the same as in the formation of a single DNA construction, i.e., a sequence specific Watson-Crick base pairing, but also unspecific base stacking forces can play a significant role. Besides, such an assembly of DNA nanostructures can be mediated in either solution or using external support surfaces, such as mica, silicon, or lipid bilayer. Nowadays lattices of even hundreds of micrometers in size have been constructed out of the self-assembled DNA tiles [40]. This is already much larger than most of the biological organelles, and could be utilized in biotechnology. This rapid development in the ability to build periodic large domains has brought new opportunities to realize large-scale functional DNA structures. Thereby, DNA arrays and lattices have been increasingly used for the self-assembly of nanometer-scale materials, ranging from nanoparticles [41] to proteins [42,43]. In addition, ordered DNA arrays could be further utilized in nanolithography to efficiently fabricate extended surfaces filled with plasmonic nanostructures [44], which could further be utilized for sensing or to form optical metamaterials. However, this would require enlargement of the structures to over millimeter scale.

This review emphasizes the importance of DNA nanotechnology as a technique to construct extended, periodic patterns out of self-assembled DNA units, i.e., tiles. We describe methods and strategies for creating a variety of two-dimensional DNA lattices. In particular, we focus on the forces involved in the self-assembly process taking place in solution as well as at the solid-liquid interface, and the delicate balance between them, which one needs to optimize for successful self-assembly. We aim to provide a general understanding of challenges and benefits in both, solution and surface assisted growth techniques, including the use of lithographic patterning processes. Besides, essential features for the generation of large-scale assemblies using tile-based assembly are discussed. The last part of the review will briefly summarize the emerging applications of DNA lattices to form novel materials by, e.g., special arrangements of gold nanoparticles (AuNP), proteins, and other components.

## 2. Planks and Nails for DNA Lattices

### 2.1. The Planks

To form a large-scale lattice of DNA structures one naturally needs suitable building blocks, a.k.a. planks, to begin with. These individual self-assembled DNA structures forming a variety of extended programmable DNA lattices through hierarchical self-assembly are often called DNA-tiles, or for short just tiles. In general, for successful self-assembly of a lattice, the structure of the tile should be more rigid than the bonding between them. As mentioned, a single ssDNA is built by the formation of covalent bonds between nucleotides; therefore, the structure remains stable during the self-assembly process. DNA-tiles, however, are bigger-self-assembled entities, usually formed by Watson-Crick base pairing between complementary bases, i.e., held together by hydrogen bonds. In this case, the connected complementary parts need to possess enough base pairs to have the melting point well above the room temperature [45,46]. In addition, one naturally needs also a clever way to organize the DNA strands into the desired form.

There are numerous ways to fabricate DNA tiles suitable for constructing lattices. Though, there are two distinguishable classes of DNA-tile structures: DNA motifs and DNA origami units, which both can serve as building blocks, i.e., tiles [39]. DNA motifs are usually small constructions comprised of several different short oligonucleotides, which corresponds to 100 to 350 base pairs (bp) per unit, and a maximum size of about ten nanometers [47]. Good examples of DNA motifs are the first branched DNA junction by Ned Seeman [9] and the famous double crossover (DX) and triple crossover (TX) tiles [48,49], which provided more rigidity compared to plain dsDNA. Using these structures for practical use, in particular for the formation of larger hierarchical patterns, has been successfully demonstrated in many studies, but has still several limitations. In addition to their relatively small size, they require high control of stoichiometry and purity of oligonucleotide strands, thus often resulting in a low yield, error-prone, and lengthy synthetic process. Therefore, a new self-assembly route was quickly adapted where DNA origami units were used as tiles forming even larger-scale assemblies.

For the first time, Rothemund proposed a different route to create DNA origami units in 2006 [11]. In his technique, a long single-stranded scaffold DNA is folded into predefined structures through hundreds of complementary short oligonucleotide chains as shown in Figure 1b. A variety of shapes can be created using the same scaffold but different sets of staple strands, thus offering a broader set of possibilities and diverse self-assembly strategies. One can easily form shapes like triangles, rectangles, or more complex objects such as smiley faces and many others each of them consisting of around 7000 bp and having the dimension of roughly 100 nm. Soon enough the method was adapted not only for 2D arrays formation but also for building hollow and multilayered 3D origami structures [12]. It allowed packing the helices into triangular, square, or hexagonal arrangements, forming bent and twisted structures [50]. The method is proven straightforward and efficient, and at the same time offers an even broader set of possibilities in the formation of larger, more complex shapes and structures. Consequently, DNA origami units were quickly adapted as excellent components for studying self-assembly and the growth mechanism of extended DNA lattices [39]. Further development of design strategies has led to new DNA constructions, which are also suitable for tiles. These include for example DNA tube circumferences [51] and ssDNA-based DNA-bricks [52], which both form lattices, as well as a huge variety of different DNA wireframe constructions [53,54,55].

### 2.2. The Nails

**Figure 2:**
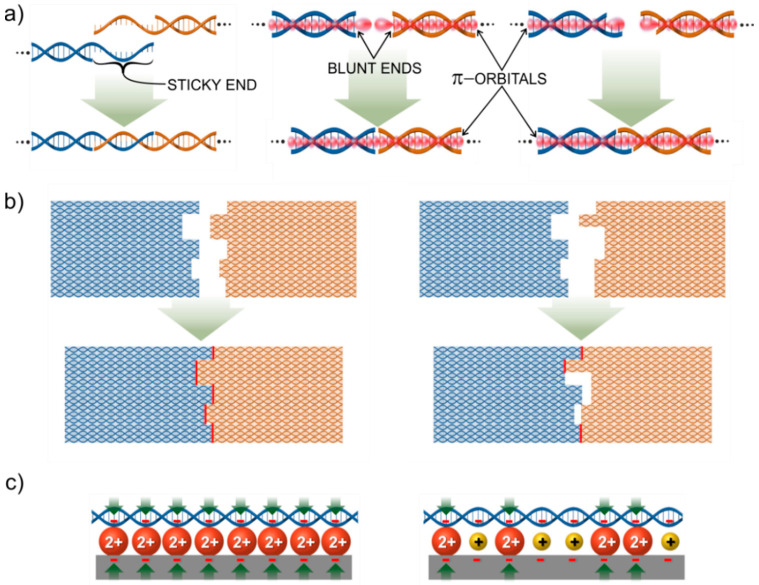
Principles of interactions between DNA-tiles: Single helix interactions are presented in (**a**) with a sticky end connection on the left. This interaction is based on base pairing between two single-stranded overhangs on different helices. The middle one illustrates the blunt end stacking, where π-orbitals of the last base pairs form a π-π connection and complete the base pair stacking through the joint. The right part shows an example of how the sticky ends and blunt end stacking can be combined. All the single helix interactions can be used in the shape complementary approach illustrated in (**b**). Bonding energy between the tiles is directly proportional to the number of successful single helix joints marked as red. In (**c**) the most common immobilization scheme of using negatively charged surface and divalent metal ions to glue the negatively charged DNA on the surface, is illustrated on the left side. The right side shows the case of the addition of monovalent ions, which occupy the positions of divalent ions and thus reduces the bonding between substrate and DNA as shown by the lower number of green arrows.

To form any larger construction out of the building blocks, or planks, they need to be joint together with some specific interactions, a.k.a. nails. The self-assembly of DNA tile can be achieved by following one of the two basic classic rules. While the first rule relies on the driving force of specific canonical, Watson-Crick base-pairing between complementary bases, i.e., A-T and G-C, the second is mediated by non-sequence-specific base stacking interactions. The early DNA motifs, like Seeman’s branched junctions [9] or DX and TX tiles [48,49], were assembled into lattices with help of so-called sticky ends, where a dsDNA helix has an ssDNA overhang on its end, called sticky end. This overhang has a specific sequence of bases that is complementary to a sticky end protruding from the other DNA helix. When these two DNA helices meet, they can form a continuous helical structure by pairing their sticky ends as shown in Figure 2a (left). Since the method is the same as the one used to assemble a tile itself, the binding energy of this kind of joint is also similar, i.e., roughly 10–20 kcal/mol per joint, depending on the number of base pairs and their type [45,46,56]. The DNA tiles usually carry several sticky ends with specific sequences matching to the sticky ends on another tile [57,58]. In general, the structure of a tile should be more rigid relative to the detachable connection between two tiles facilitating the self-assembly of larger constructs. Sticky end hybridization can be also incorporated in other geometric designs. For example, it can be used to connect DNA tiles by connecting adjacent helices side-by-side as demonstrated by the group of Shalom Wind [59].

The second driving force, i.e., blunt end stacking, uses the stacking interaction at blunt ends of dsDNA helix structures. Inside a dsDNA helix, the base pairs are bound not just by the backbone of the DNA, but also the connection is enhanced via stacking interaction due to their overlapping π-orbitals, thus forming π-π interaction between the bases. The base pairs at the ends of the helix have unpaired π-orbitals sticking out of the helix. This can establish π-π interaction with the terminal bases of another dsDNA helix as shown in Figure 2a (middle). The strength of the interaction between two DNA-tiles having blunt ends can be controlled by the number of adjacent helices and the base sequence of the end [11]. Today, base stacking interactions are widely used as directional forces in hierarchical surface-mediated self-assembly of DNA origami units. The blunt end stacking is much weaker than sticky ends and unspecific for a choice of bases. The binding energy for a triple stacking has been measured to be about 8.6 kcal/mol [60].

To fine-tune the binding energies between the helices and tiles, the above two rules can be combined as shown in Figure 2a (right) [61]. Here, the helices have too short sticky ends, i.e., one to three bases, to hold them together. However, when combined with the blunt end stacking between the base pairs at the ends the total binding energy of the joint becomes high enough to sustain the interaction, i.e., the intermediate force between the plain blunt end and a proper sticky end.

Shape complementarity can be additionally implemented in the design of the tiles and thus combined with the blunt end stacking and/or sticky-end interactions. It enhances the directional control of the self-assembly process because the connection between complementary shapes is much stronger than for a non-matching pair. This is illustrated in Figure 2b, where the formed blunt end π-π stackings are highlighted by red for matching and non-matching tiles. Shape matching can also be used for arranging higher-hierarchy systems as demonstrated by the research group of Endo [62]. Even so, often, the combination of inter-origami binding forces, i.e., sticky and blunt end interactions, in addition to some more exotic bondings, such as Hoogsten bonding is needed to achieve directional control of hierarchical origami assemblies. Combining different types of connections can facilitate the fabrication of even kinematic DNA structures comprising multiple origami units with well-defined moving ranges and routes [63]. This is, however, out of scope of this review, where we concentrate on large-scale static lattices.

### 2.3. Assembly Protocols

If the lattice out of DNA-tiles is first formed in a solution and only after that transferred onto a substrate, the forming lattice is constantly subjected to the strong drag and shear forces present in the solution. From the above-discussed forces between the tiles, the sticky end hybridization is strong enough to govern the drag forces. However, plain blunt end stacking is not usually strong enough and the feasibility of the combined connections depends strongly on the total geometry, and number of the joins. Though, nowadays people are keen on using surface-mediated self-assembly approaches, where the tiles weakly bind to the substrate, and can still diffuse on it, rearrange, and consequently form a lattice. In this case, all the above connection rules are feasible, since the substrate provides additional support. However, it is very important to also control the binding energy between the DNA-tile and the substrate. The most common way to attach DNA-structures on a substrate is to use a freshly cleaved mica surface, in a presence of divalent metallic ions, such as Mg^2+^, which are already contained in the origami folding buffer, bridging the DNA and substrate together as shown in Figure 2c. Even though the attraction forces between DNA tiles and surface are rather weak, still they can partially impair the desired diffusion on it. Yet, one can fine-tune this interaction by adding monovalent ions, like Na^+^, which weaken the interaction by partially replacing divalent ions from the surface, forming a more diffuse charge layer. As a result, DNA structures become more mobile and start rearranging on the surface forming a lattice [64,65,66].

The four main approaches can be distinguished in self-assembling DNA-tiles into target structures. Among them is: one-pot “*mix and go*” -method, which is a key concept of DNA nanotechnology, inspired by Seeman [49,67,68]. In this method, each tile of the set contains unique connections, usually sticky ends, leading the tile into the desired position during self-assembly, but above all the final large lattice is formed in the same process, and thus within the same solution, in which the tiles are formed. The second method, *hierarchical self-assembly*, is usually a two-step process where a specific group of tiles is pre-assembled in test tubes and then afterwards combined to form a larger structure [12,69,70,71,72]. In this approach, the combination of sticky and blunt end/shape complementary interaction forces are often employed, as well as a supporting substrate. In the more sophisticated *algorithmic self-assembly*, tiles are programmed with specific binding domains, which can explicitly bind to another unit following an algorithmic rule. This often leads to a fully programmed lattice whose size is also fixed [73,74,75,76,77]. The last distinguished method is called *scaffolded tile-assembly*, where a longer strand is used as a scaffold upon which DNA-tiles attach to form various patterns. The “scaffolded frames” procedure often takes advantage of all the above-mentioned interaction forces, in addition to surface supports interaction stabilizing the self-assembly process [78].

## 3. Assembly of DNA Lattices in Solution

Many solution-based lattice assembly strategies have been reported in literature yielding two- and three-dimensional DNA nanostructure lattices. Originally the solution-based assembly was introduced by Seeman as a one-pot “*mix and go*” -procedure [9], which remained the main method at the beginning of the structural DNA development, until the more sophisticated ones like hierarchical self-assembly were introduced. Hierarchical self-assembly is a compelling approach to create structurally versatile, regular DNA patterns with a dimension beyond a 1 µm limit. Many finite-two- and three-dimensional DNA or infinite-crystal-like structures have been successfully assembled following this approach [67,79,80,81,82]. Generally, in solution, the assembly is mostly driven by sticky end associations connecting the structures, as the blunt end interaction is usually not strong enough to hold the larger assemblies together. The selectivity of the tile-to-tile interactions can be further enhanced by utilizing shape complementarity at the ends of the nanostructures. Nevertheless, the challenge still remains in the optimization of assembly parameters including annealing conditions to obtain structures at high yield. In addition, the deposition of solution-assembled lattice onto a substrate without breaking is not a trivial task.

### 3.1. Lattices by Early DNA Motif

The essential foundation of DNA nanotechnology is Nadrian Seeman’s iconic design of complementary sticky end and branched DNA junction to make geometric objects and periodic lattices [9,83]. His design became the most common self-assembly method in the DNA nanotechnology called, multi-arm approach. In this approach, DNA sequences are designed to assemble into branch motifs with unique, complementary sticky ends. The sticky ends precisely guide the inter-tile interaction and position of the tile in the assembly (Figure 1a). Following this methodology, Seeman and coworkers created various DNA motifs that could self-assemble into higher-order DNA arrays [84,85,86]. However, multi-arm junction design suffered from geometric instability and flexibility, and that often resulted in poor structural predictability. Hence, ultimately, they were not suitable for the assembly of extended patterns.

**Figure 3:**
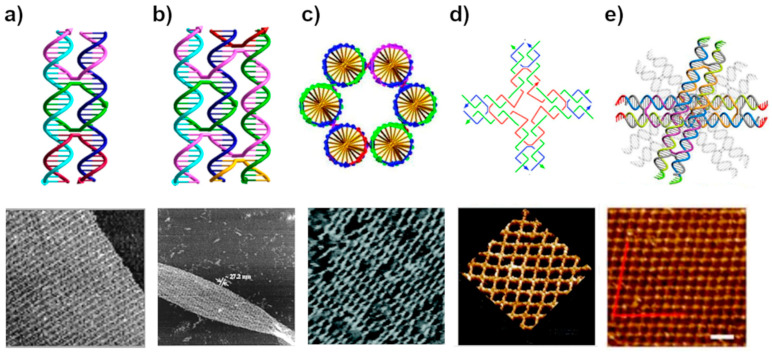
The early DNA tile motifs and AFM images of their corresponding 2D lattice assemblies. (**a**) DX DNA tile comprises two parallel helices connected by the ssDNA strands (pink, green, and red) crossing to the other helix at the crossover points [68,96]. (**b**) TX DNA tile is formed similar to the DX tile, but now the ssDNA strands connect three helices together. The pink strand is even part of all the three helices [49,96]. (**c**) Six-helix bundle DNA tile, where all the six helices are connected to their two neighbors by crossovers, finally forming a tube-like structure [91]. (**d**) Cross-shaped tile formed from branched DX-tile or four DX-tiles joined together at one end [79]. Adapted with permission from AAAS. (**e**) Layered crossover DNA tile [97]. Adapted with permission from [68] copyright 1999, [49] copyright 2000, [91] copyright 2005, [97] copyright 2018 American Chemical Society.

To overcome this problem, a new motif design was constructed by joining two parallelly-oriented double helices by strands crossings between them at two crossover points, which led to the formation of a branched complex called DNA double crossover (DX) molecule (Figure 3a) [48]. The DX motif resulted in a more rigid nanostructure compared to linear DNA, and for that reason, it was officially named as the “DNA tile” unit for the first time. In Figure 3a, the successful construction of a first periodic 2D crystalline lattice for the DX motif is presented [67]. The arrays were formed by mixing two to four different DX tiles to display a striped pattern. Later the DX-tile was used for example to produce Sierpinski triangles via algorithmic self-assembly [74], and fully addressable finite-size tile-blocks [87]. Soon after the invention of the DX-tile, the design was extended to the DNA triple crossover complex (TX), (Figure 3b) [49], which was used to produce a variety of self-assembled linear arrays [88], 2D lattices [49], and DNA tubes [89]. LaBean et al. [90] increased the rigidity of the TX-tile even more by designing a circular form of it called three-helix bundle. Almost simultaneously, Seeman et al. [91] also introduced another tubular DNA-tile called six-helix bundle shown in Figure 3c, which become widely used later [92,93,94,95].

Another example of tiles referred to as a “cross tile” was introduced by Yan et al. [79] in 2003, shown in Figure 3d. Thanks to its symmetric structure, it assembles into well-formed 2D-lattices. Consequently, a variety of DNA tiles, including rigid cross-shaped, three point-star, or six point-star motifs tiles were designed and assembled into large 2D arrays with square or hexagonal and triangular cavities, respectively [70,72,98,99,100]. In addition, some finite size addressable lattices were also designed [69] Most of these designs followed a two-step hierarchical assembly, where each tile is formed separately and then they are combined together. In recent studies, Yan et al. developed a derivative from the traditional double-crossover DNA motif, named layered-crossover tile to construct a set of rhombus-like layered-crossover DNA lattices. In the motif design, two or four-layered crossover tiles bridge neighboring layers with predetermined orientation [97]. Similarly, Qian and coworkers created a triangular tile that could form large 2D arrays and 3D shapes approximating a rhombic triacontahedron [101].

Although the DX and TX tiles have proven to be fully addressable assembly method, the number of required strands and the costs increase linearly with the number of unique tiles. Further developments of similar design strategies have been reported including the paranemic crossover (PX) motif [102], which brought inspiration for nanoconstruction of 3D objects [102], and multihelical bundles [90,91,103] or parallelogram DNA tiles [104] that self-assemble into linear or 2D arrays and 2D lattices with diamond-shaped cavities [102]. Even though a multi-arm approach is still a method of choice to efficiently construct crystalline-like structures it requires high control of stoichiometry and purity of oligonucleotide strands, thus often resulting in low yield, error-prone, and lengthy synthetic process. Therefore, a new self-assembly route was quickly adapted where DNA origami units were used as tiles forming even larger-scale assemblies.

### 3.2. DNA Origami-Tile Assembly

**Figure 4:**
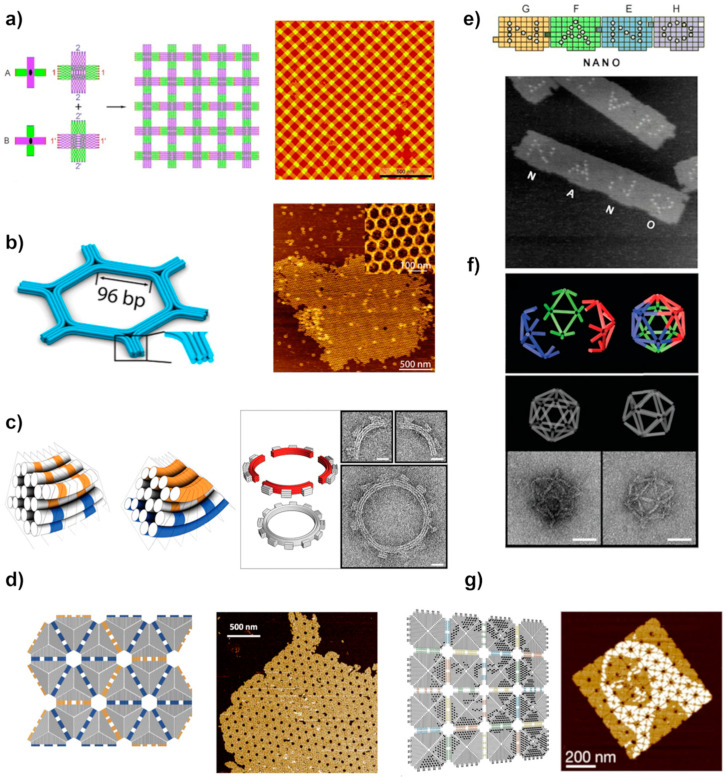
DNA structures and lattices formed from DNA origami tiles through hierarchical self-assembly. (**a**) 2D lattice assembled from cross-shaped tiles by sticky end hybridization [34]. (**b**) 2D hexagonal lattice assembled from hexagonal DNA origami tiles [61]. (**c**) Design of a DNA bundle modified to bend into a quarter circle, which are subsequently joined together to form a gear; scale bars 20 nm [50]. Adapted with permission from AAAS. (**d**) The design of self-assembled structure out of the asymmetric tiles and the corresponding AFM image [101]. (**e**) Jigsaw DNA origami units; although sticky end hybridization controls the alignment of the pieces, base stacking and shape complementarity reinforce the binding; image size 525 × 525 nm [105]. (**f**) Formation of large DNA cages “icosahedron” assembled three different wireframe type subunits; scale bar 100 nm. Adapted with permission from [12]. Copyright 2006 Springer: Nature. (**g**) Design of DNA arrays with a Mona Lisa pattern and corresponding AFM image. Adapted with permission from [110]. Copyright 2017 Springer: Nature. Adapted with permission from [61] copyright 2016, [101] copyright 2018, [91] copyright 2005, [97] copyright 2018 American Chemical Society.

An alternative method enabling the formation of larger, infinite crystal-like lattices or more complex, finite structures is the hierarchical self-assembly of DNA origami. Similar to the DNA motif, DNA origami can act as a tile unit and thereby self-assemble relying on sticky ends or other interactions presented in Section 2. Correspondingly, these interactions can be enhanced by applying the complementary matching shapes improving the tile-to-tile interactions, especially their selectivity, as shown in Figure 2b. The first experimental demonstrations of large hierarchical assemblies from DNA origami were a gear formed by the association of four quarter circles shaped origami (Figure 4c) [50], an icosahedron made from mixing three wireframe style monomers (Figure 4f) [12], and an infinite crystalline 2D lattice of two origami tiles propagating into two independent directions (Figure 4a) [34,105].

The large dimension and complex molecular dynamics of DNA origami have a profound effect on the final design of a structure and dictate the interaction between the origami units. While designing a 2D DNA origami structure, a global twist with respect to a tile plane needs to be taken into account [106]. This structural distortion is a consequence of the conformation design of DNA origami with an average twist of 10.67 bp per turn, which slightly deviates from the natural B-type helical twist of 10.5 bp per turn. The small increase accumulates when hundreds of units are assembled together. To overcome the problem, Liu et al. [32] constructed a set of rectangular shape origami arranged in a zigzag ribbon pattern to avoid distortions and investigated the self-assembly of higher-order structures. They also found that the position and strength of the connecting strands between the linked units have a strong influence on the quality of the final assembly product. Even in some cases, it led to the assembly of completely different structures [33].

Large origami structures contain a significant number of DNA helixes and in many cases, these have their ends side by side at the edges of the origami. Therefore, base stacking interaction forces by many parallel blunt ends can easily hold the origami units together [107]. Nevertheless, the solution-assembled larger structures are often fragile and easy to break. Therefore, instead of base stacking interaction, sticky ends hybridization is commonly used to ensure structural support and control over the assembly. The studies have shown that the combination of both interactions improves control over the various pattern assemblies. Qian and coworkers modified triangular DNA with both interactions to form large 2D arrays and 3D shapes approximating a rhombic triacontahedron (Figure 4d) [101]. Figure 4e represents a classic example of the formation of large planar structures made of DNA jigsaw pieces where the combination of different types of forces improves the control of a whole system [105]. The inter-unit hybridization and stacking at the interfaces of shape-complementary edges allowed regioselective origami pairing [107,108,109]. Wang et al. assembled a more complex, micron-scale honeycomb lattice from a set of hexagonal DNA origami tiles as illustrated in Figure 4b. The hexagonal DNA origami tiles were self-assembled through a combination of connector strand design, base hybridization, and blunt end interaction. Later, they used a pre-assembled honeycomb lattice as a platform for the positioning of nanoparticles (NPs) [61].

The aforementioned approaches usually lead to the assembly of relatively simple patterns. To increase complexity and range of the scale one needs to implement algorithmic self-assembly [73,74,75]. Complex programmable features have been achieved using DNA tiles leading to high yields of superstructures of micrometer dimensions [73,74,75,76]. The fractal assembly allows scaling up the complexity of DNA nanostructures with arbitrary patterns [77]. For example, Tikhomirov et al. [110] used this approach to generate patterns in the shape of a famous painting by Leonardo da Vinci, Mona Lisa, as shown in Figure 4g. Following the same approach, Gang et al. [111] designed a planar DNA origami frame and used it to periodically organize gold nanoparticles, creating diverse 2D architectures.

## 4. Surface-Assisted Assembly

As mentioned in the previous chapter, the self-assembly of an extended lattice in the solution is limited, because the whole structure is exposed to the drag and shear forces induced by the solvent. These forces increase with the dimensions of the lattice and thus limit the conceivable size. Therefore, in almost all the lattices formed in a solution, sticky ends have been utilized as the main driving force ensuring the connection between individual tiles. Another challenge remains during deposition of the pre-assembled lattice onto substrate surface. Many times, it results in structural deformations along with some damages or torn structures—the larger the structure the lower the probability for a successful and error-free deposition. Because of these reasons and with the emerging trend towards larger origami-based lattices, researchers came up with a new strategy, where the prefabricated tiles are adhered on a surface and the assembly of the lattice happens by controlling the diffusion of the tiles on it [64,71]. In surface-assisted assembly, the substrate provides needed support and route to more stable and reproducible patterning achieving larger lattices.

The large surface area and thus highly negative charge of origami units promote the electrostatic hierarchical self-assembly on external surfaces. In addition to the tile-to-tile interactions, discussed above, organizing DNA nanostructures on a surface requires an understanding of the role of the three main factors controlling the diffusions of origami tiles on the substrate, namely DNA-surface interaction, DNA concentration, and the assembly time. The type and number of components making up the system affect the type of acting forces, their strength, and the energetic pathways towards the most stable structure. The function of the substrate surface is also crucial, given the fact that the mobility of the DNA units on the substrate is one of the key prerequisites for the lattice assembly. Therefore, the assembly process typically requires weak adsorption conditions to ensure the DNA surface mobility. The adsorption forces, however, should be tuned as such to still provide required attraction between the surface and DNA tiles to keep the already attached units together. Surface support is an important factor in obtaining large scale-assemblies because it stabilizes the DNA-DNA interactions, and confines the structures within physical space, thus boosting the probability for the DNA units to meet and initiate binding.

Even though the precise design of DNA structures and spatial organization of the objects with nanoscale accuracy has been accomplished and monitored in real-time, it is still challenging to obtain large periodic lattices. Many of the formed lattices still suffer from assembly defects, low yield, and small dimensions. In the following section, we go through feasible ways to create extended DNA patterns, particularly with the help of cationic compounds through hierarchical assembly on mica and lipid bilayer membranes used as surface supports. In addition, we discuss about template-based assembly on silicon and other surfaces.

### 4.1. Mica

Mica surfaces are the most common solid support for DNA origami studies due to their inherent advantages. Besides the low cost, mica features relatively large atomically flat areas and requires simple sample treatment procedures. Mica has a mineral net-like structure, containing an octahedral complex aluminum layer sandwiched between two tetrahedral SiO-layers [112]. When it comes in contact with water, potassium ion, which naturally bridge the ionic mineral sheet of mica, dissociates from the surface, leaving the unbalanced negative charge. Like the mica surface, DNA origami structures are negatively charged; hence, the adsorption on the mica surface is initiated by cations stabilizing DNA-mica interaction as discussed above in Section 2. The buffer in which origami is dissolved usually contains an Mg^2+^ counterion mediating the electrostatic attraction. Surface-assisted self-assembly also requires a suitable interaction between DNA units to form larger assemblies. On mica, the stacking interaction between blunt-ended origami is already capable of holding structures together, forming 1D or 2D arrays.

One of the first attempts to arrange DNA tile structures into periodic patterns was accomplished with pre-formed, branched star-shaped motifs. These relatively small structures were immobilized on a mica surface and incubated at 50 °C [71]. Based on this study, the DNA motifs-mica surface interaction confines the tiles, restricting their flexibility, and increases the interaction between them. Additionally, similar studies have revealed that the 2D assembly kinetics is relatively fast in contrast to the previously reported sticky-end mediated-assembly [66]. The direct observation of the processes such as the adsorption/desorption of the DNA and its diffusion on the surface would deepen the understanding of kinetics at the solid-liquid interface. High-speed AFM is a method of choice to investigate the morphology and dynamics of lattice formation [113,114]. Despite relatively straightforward assembly protocol, the real challenge remains in understanding the requirements and precise condition in regulating the self-assembly of the DNA-tiles leading to the large single-layer patterns.

**Figure 5:**
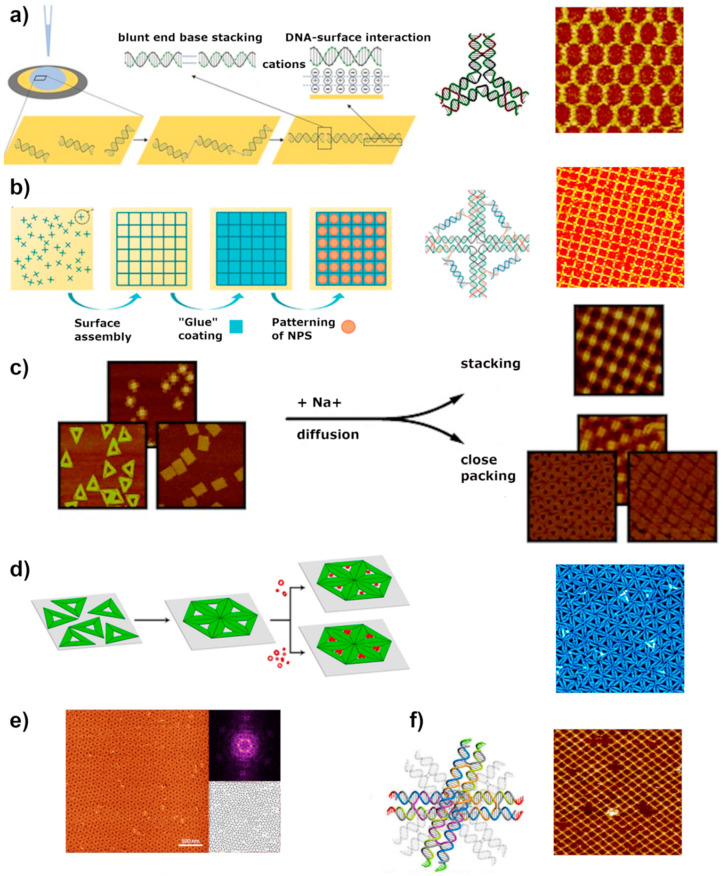
Surface-assisted DNA origami self-assembly on mica. (**a**) DNA molecules loosely adsorb on the surface forming salt bridges with the mica surface, after which they rearrange themselves to maximize DNA-DNA blunt end stacking interaction [66]. (**b**) Self-assembly process of 4-point star motifs into a 2D lattice, which is later used for patterning NPs in the presence of Ni^2+^ to glue the lattice to mica [115]. (**c**) The self-assembly of triangles and rectangles is mediated by reaching an electrostatic balance between the DNA nanostructures and mica surface. Non-interacting triangular structures assemble into close-packed structures, while attractive blunt-end stacking interactions between the cross-tiles lead to the extended 2D lattice assembly [64]. (**d**) Triangular origami tiles assembly at high Na^+^ concentration [116]. (**e**) AFM image of a highly ordered lattice of triangular origami tiles as well as corresponding FFT and Delaunay triangulation. The lattice was self-assembled within a buffer with Ca^2+^/Na^+^ ions during 90 min incubation [40]. (**f**) Design of layered-crossover tile and a corresponding image of a self-assembled 2D crystal structure [97]. Adapted with permission from [115] copyright 2019, [116] copyright 2016, [97] copyright 2018 American Chemical Society.

The large surface area and negative charge of DNA structures enable control of the adsorption forces by exploiting the competition between different ions. The effects of various ions allow tuning the forces and promote the formation of extended patterns. Divalent cations promote the immobilization of DNA-tiles on the surface, by forming salt bridges between the surface and structures, as discussed in Section 2 and shown in Figure 2c. One of the advantages of surface-assisted methods is that by tuning the divalent cation concentration, the integrity and homogeneity of the lattice can be achieved. Typically, the immobilization of DNA origami requires magnesium concentration between 10 and 200 mM to overcome the repulsion barrier and bridge the surface and structures. Too low concentration can lead to structural instability, whereas the addition of too high concentration results in too strong adsorption of DNA structures reducing surface mobility. Recently, Liu and colleagues demonstrated that the homogeneity and integrity of a tetragonal array’s formation can be achieved only by tuning the concentration of Mg^2+^ ions and exploiting blunt stacking interaction between blunt ends. (Figure 5b) [115].

Even though magnesium is the most commonly used divalent cation to bridge the origami structures and mica surface together, other metals play a critical role in the origami adsorption onto the surface. In particular, transition metals have been used as a form of glue to preserve the structures on the surface. Mao group studied the influence of Ni^2+^ ions on 2D arrays formation, shown in Figure 5a [66]. The experiment showed that even a small quantity of nickel cations is sufficient to stop the diffusion and “freeze” the origami structures on the mica substrate [65]. Since Ni^2+^ ions bind generally stronger to DNA structures, a high concentration of metal may lead to structural deformation.

Once DNA tiles are adsorbed onto the mica surface loosely enough, base stacking interaction between blunt ends of DNA units enables the rearrangement of the structures on the surface, to maximize the interactions between them. For successful self-assembly, the strength of DNA-DNA and DNA-surface interactions need to be comparable [66]. The electrostatic balance between surface and DNA structures can be reached by the addition of competing monovalent cations. For instance, the addition of sodium ion reduces the number of salt bridges, displacing Mg^2+^ ions from the interface, directly affecting the surface mobility of origami structures, as shown above in Figure 2c [114]. The first successful assembly of large-scale ordered lattice on mica surface was accomplished by Rafat et al., demonstrated in Figure 5c. In their work, the addition of sodium ion weakened the interaction by partly replacing magnesium ion, thus forming a more diffusive charged layer eventually leading to lattice self-assembly [64].

One of the assembly approaches is to only optimize the DNA tile concentration and mobility on the mica surface and pack the surface so densely that it results in macroscopic surface area, homogeneously covered with formed lattice [66]. This very straightforward approach does not require any specific design and optimization of molecular forces. Ramakrishnan et al. [116] created a monolayer of densely packed triangular origami tiles following the same, cation-mediated assembly (Figure 5d). At a Na^+^ concentration of 200 mM, that is, 20-fold excess over Mg^2+^, a densely packed monolayer with hexagonal symmetry was formed. Keller group also conducted a comprehensive study on the lattice formation by investigating the impact of sodium concentration in the 10 mM Mg^2+^-containing DNA origami buffer [114]. Notably, they suggested that the Na^+^/Mg^2+^ ratio has a similar effect on lattice assembly dynamics as a substrate temperature in thin-film growth. The assembly of triangular DNA origami structures into highly ordered lattices was best achieved at 75 mM concentration of sodium cations, i.e., 7.5-fold excess over Mg^2+^. Although the assembled lattices did not feature significant dislocation, some minor defects were still observed. Longer incubation times increase lattice quality and order, as expected because the triangular structures had more time to organize into a well-ordered structure. Nonetheless, the assembled lattices are usually poly-crystalline with crystalline domains having a random orientation to each other [117]. Lately, Hong et al. [97,118] studied the assembly of layered-crossover tiles and found out that in the case of surface-mediated assembly, the single-layer of DNA 2D crystal fully covered the mica surface even up to several microns by only adjusting the origami concentration while keeping the monovalent cation constant (Figure 5f).

The essence of the assembly process is to control the interplay between ionic strength and electrostatic interaction at the liquid-surface interface. Ionic strength is determined by the size and hydration energy of an ion [119]. Among alkali metals (1A group), K^+^, Na^+^, and Li^+^ ions bind mainly to the DNA backbone phosphates stabilizing the DNA duplex. In terms of binding activity to DNA, potassium ion is favored having the largest diameter and lowest dehydration energy. Lately, the Keller group reported a detailed topological analysis of highly ordered DNA origami lattice formation under the influence of different ions [40]. Remarkably, they found that even though Li^+^ ions similarly interact with DNA as Na^+^ ions, the hydration and adsorption behavior is different, due to their smaller radius. They further concluded that Na^+^ ion is more effective than Li^+^, in terms of replacing Mg^2+^ and thereby promoting the origami assembly. Potassium, however, with a similar hydration radius showed distinctively different results [40]. Under potassium influence, the origami structures either remained adsorbed on the mica surface or started to build-up into DNA multilayers. This behavior could be explained by the reduction of the electrostatic repulsion between the negatively charged DNA tiles.

The binding of alkaline earth metals (2A group), to the phosphate backbone of the DNA follows the same trend as for alkali metal ions. The binding energy gradually weakens from Mg^2+^ to Ba^2+^ ions [120]. Keller et al. [40] reported that Ca^2+^ ions can be displaced more easily from the backbone phosphate than Mg^2+^ ions resulting in more efficient lattice formation, as shown in Figure 5e. Transition metals instead, bind preferably to the nucleobases rather than phosphate backbones with binding affinity following Hg^2+^ > Cu^2+^ > Cd^2+^ > Zn^2+^ > Mn^2+^ > Ni^2+^, Co^2+^ > Fe^2+^. Transition metals in general result in stronger binding than metals in 1A and 2A groups [119]. For instance, Zn^2+^, Ni^2+^, and Co^2+^ ions can effectively enhance DNA adsorption to the mica surface. However, due to their strong interactions not only with the mica surface but also with DNA structures, the transition metal ions are more difficult to exchange with monovalent cations to promote the higher diffusion rate of DNA origami assembly [121,122,123].

Also, the symmetry of origami building blocks has been shown to affect the assembly efficiency in addition to the structure’s final geometry. DNA structures featuring inert edges and regular shapes such as triangles or rectangles were prone to associate into close-packed structures, whereas blunt-ended origami structures mainly lead to extended 2D lattices [64]. Recent studies on the dynamics of lattice formation performed by the Keller group found that introducing impurities in a form of rectangular DNA origami to the pre-assembled hexagonal lattices did not cause any defects in the structure. They concluded that rectangular and triangular origami structures of the same size have very different mechanical properties. DNA origami triangle is rigid with preferential adsorption shape, whereas a twist-corrected rectangular are rather flat and structurally flexible [117].

### 4.2. Lipid Membrane

**Figure 6:**
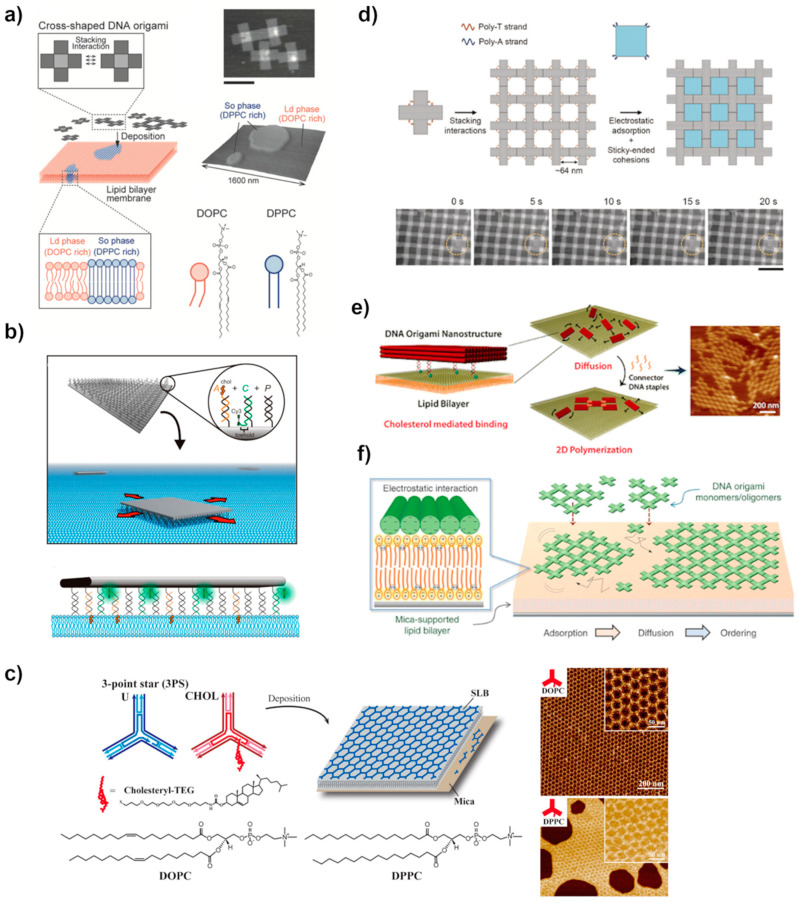
Self-assembly of DNA origami structures on mica-supported lipid bilayers surface. (**a**) cross-shaped DNA origami assembly on a phase-separated lipid bilayer-membranes formed in the presence of DOPC and DPPC molecules, scale bar: 100 nm; the structure and schematics of DOPC and DPPC molecules [125]. (**b**) Design and interaction of DNA-cholesterol barges with a support lipid bilayer. A rectangular DNA origami bearing unlabeled Passenger ssDNA strands (P) on one side, permitting the attachment of cholesterol-labeled ssDNA Anchor (A) and/or fluorescently labeled y3-labeled Cargo strands (C) to the origami via hybridization [126]. (**c**) Cholesterol-TEG (CHOL) modified 3-point star tiles assembly on DOPC and DPPC bilayers [128]. (**d**) Assembly of DNA framework from cross-shaped origami tiles through base stacking interaction. Square-shaped origami is trapped in the cavities of the framework by increasing Mg^2+^ concentration or via sticky-end hybridization [62]. (**e**) Visualization of DNA block structures adsorption on the lipid membrane via hydrophobic cholesterol anchors [129]. (**f**) The self-assembly process on mica supported lipid bilayers by Mg^2+^-mediated electrostatic binding [42]. Adapted with permission from [126] copyright 2014, [128] copyright 2017, [129] copyright 2015 American Chemical Society.

The hierarchical organization of DNA components into extended structures has been achieved also on mica supported lipid bilayer (SLB) membranes [124]. These are highly ordered two-dimensional materials formed by self-association of DOPC (1,2-dioleoyl-sn-glycero-3-phosphatidylcholine) and DPPC (1,2-dipalmitoylsn-glycero-3-phosphatidylcholine) synthetic zwitterionic lipids. The whole structure is shown in Figure 6a [125]. Both lipids contain the same head group (phosphocholine, PC) but they exhibit slightly different phase behaviors [125]. DOPC, the unsaturated variant, exists as a fluid, thus forming a liquid-phase whereas DPPC, the saturated variant, exists in a solid form, thus creating the gel-phase. The studies have shown that the self-assembly process of DNA origami on top of these SLBs strongly depends on the fluidity and charge density of the bilayer surface. The DNA structures preferentially bind to the liquid-phase forming 2D lattices on that, as opposed to a gel-phase where only aggregates were formed [125,126]. Recent studies by Kempter et al. [127] supported these findings and additionally pointed out that surface roughness and flexibility are also critical factors for the immobilization of origami structures, affecting their diffusional behavior. The regions with high-surface curvature or defects of the membranes resulted in a high-fraction of immobile DNA structures.

The self-assembly on a lipid membrane is based on a conceptually similar approach as in the case of the assembly on mica, i.e., mediated by electrostatic interaction and enhanced surface diffusion mobility at lipid bilayer [42,127]. In this case, the interactions between negatively charged DNA backbone and polar or charged lipid head group in the presence of divalent ions, such as Mg^2+^, are sufficient to promote membrane adherence. Suzuki et al. [42] self-assembled cross-shaped tiles, similar to Rafat et al. [64] with blunt ends into the large 2D crystal at the DOPC lipid bilayer (Figure 6f). According to their studies, Mg^2+^-mediated adsorption of DNA structure on the SLB membrane is sufficient to retain mobility leading to the assembly of the ordered crystal structure. In the same studies, they tested the effect of the addition of monovalent ions on the preformed lattices [42]. Interestingly, the addition of monovalent ions, such as Na^+^ or K^+^ changed the ionic strength of the binding and led to the reversible detachment of the whole structure from the surface [42]. In the follow-up studies, Suzuki et al. [62] succeeded in the formation of an even more complex assembly, where a pre-assembled DNA framework from cross-shape origami structures was further advanced by attaching origami structures with the shape and size fitting directly to the cavities shown in Figure 6d. Construction of such an assembly was achieved by tuning the Mg^2+^ concentration and lipid composition.

Lately, Kempter et al. [127] demonstrated that by tuning the ionic strength of the solution the diffusion rate of the origami can be well-controlled. Already at the 5 mM of Mg^2+^ and 0 mM Na^+^, the surface diffusion was completely stopped on glass SLB membrane. They later found out that this behavior is affected by the type of substrate that supported the SLBs. On the mica-supported lipid bilayer, the diffusion only decreased when the monovalent ion was removed, however, any attempt to transiently stop the origami diffusion, even with the increased magnesium concentration up to 50 mM were not successful. One explanation contributing to the unexpected behavior of DNA structures on glass is the direct charge interaction between the glass surface and origami. Apparently, at a certain magnesium concentration, the charge inversion can occur only at the glass surface mediating the direct binding of positively charged glass and negatively charged origami. Notably, it was observed that a high concentration of divalent ions contributes to the reduction of membrane thickness, which can also somewhat account for the diffusional behavior of DNA nanostructures on the SiO_2_ surface.

In addition to the above purely electrostatic adsorption, DNA tiles can be also attached to the lipid bilayer membrane by functionalization of the DNA structures with hydrophobic moieties e.g., cholesterol, porphyrin, or ethyl phosphorothioate [25,130,131]. Typically, DNA tiles are equipped with protruding oligo strands, i.e., sticky ends, which are further hybridized with oligonucleotides labeled with hydrophobic lipid molecules that are embedded in the lipid membrane [126,128,129]. For example, Kocabey et al. [129] utilized this idea to assemble large multilayer arrays of DNA bricks functionalized with cholesterol to enhance the interaction with phospholipid membranes (Figure 6e). Johnson-Buck et al. [126] also used a similar set-up to anchor rectangular DNA origami to the lipid bilayers by conjugating them with cholesterol strands, as explained in Figure 6b. The cholesterol labels induce stable association with the supported lipid bilayers but still allow lateral diffusion on it. Additionally, the interaction between the cholesterol-labeled DNA origami and lipid bilayer can be modulated by adjusting the number and distribution of the chemical groups, i.e., altering the size and number of cholesterol modification [126,129]. Smaller DNA motifs have also been organized on the membranes. Avakyan et al. [128] demonstrated that the combination of blunt-end stacking interaction, cholesterol-mediated DNA anchoring, and electrostatic binding to a lipid bilayer can lead to the assembly of micrometer-sized 2D DNA tile arrays (Figure 6c). They found that cholesterol-TEG (CHOL) modified tiles on liquid-like DOPC bilayer formed extensive hexagonal arrays, in comparison to unmodified tiles (U), where the structures did not bind at all. The same behavior was observed also by others [126]. Surprisingly, on the gel-like DPPC bilayer, both types of tile assemble into lattices, in the presence of Mg^2+^ cations.

An interesting alternative to link two DNA units together was presented through photoresponsive DNA origami nanostructures on SLBs [124]. The azobenzenes modification on the outer edge of the hexagonal-shaped DNA origami structure controlled the assembly and disassembly of the dimer pair. Under photo-irradiation, the azobenzene moieties can form a duplex in *trans*-form and dissociate to *cis*-form, resulting in reversible assembly and disassembly process.

### 4.3. Templated Assembly (on Silicon and Other Surfaces)

In the past, various techniques have been employed to achieve precise control over the position of DNA tiles in periodic structures. As presented in the previous sections, successful formation of micrometer-sized structures in test tubes and on solid substrates was achieved by controlling the electrostatic forces, hybridization between DNA units, and optimal annealing conditions, etc. However, the arrangement of individual DNA units into organized patterns is often challenging resulting always in some amounts of structural defects. An alternative method for surface-mediated DNA assembly relies on the confinement of preassembled DNA structures into lithographically patterned cavities or attachment pads. This technique is mostly based on top-down lithographic patterning techniques that use physical or chemical processes to fabricate patterns on a surface with a nanometer resolution. Combining both techniques does not only improve the resolution of lithographic approaches down to the nanometer scale but also resolves some of the challenges related to the bottom-up arrangement of individual DNA units. In this section, we will focus on the combination of these two techniques to create periodic DNA structures.

In general, nanolithography comprises numerous top-down techniques that use various methods, typically a focused beam of electrons or light, to create the desired pattern on an electron/photosensitive film, called a resist, and subsequently transfer this pattern to the underneath substrate by some type of deposition or etching. The substrate is typically covered with a hydrophobic material that becomes hydrophilic upon the O_2_ or plasma treatment. The shape and size of the formed attachment pads are typically created to match the shape and size of the origami units. Besides, the method offers the convenience to tune the position and spacing between the docked DNA objects, allowing for example an easy way of investigation of optical properties of plasmonic lattices that depend on the periodicity.

**Figure 7:**
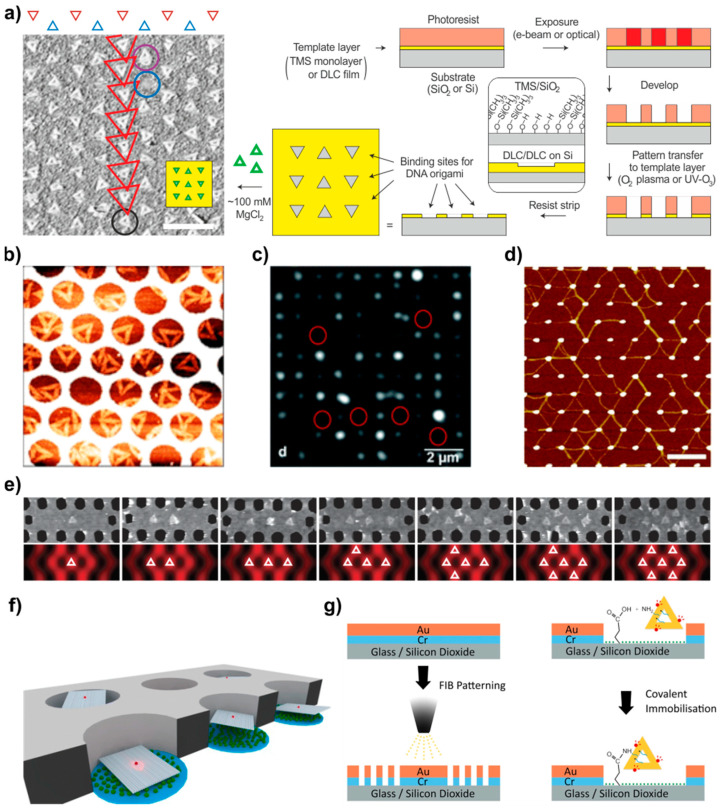
DNA origami immobilization on the templated surfaces. (**a**) AFM image of DNA triangles and the patterning process of the DNA binding sites; scale bar 500 nm. Adapted by permission from Springer: Nature Nanotechnology [132], copyright 2009. (**b**) AFM image of DNA triangles in 160 nm diameter nanoholes created by nanoimprint lithography [138]. (**c**) Total internal reflection fluorescence micrograph of porphyrin-modified DNA origami attached to the e-beam treated Teflon AF nanopillars. Red circles represent empty slots, without attachment. Adapted from Ref. [140] with permission from The Royal Society of Chemistry. (**d**) AFM image of DNA origami tubes with thiol-modified ends connecting a hexagonal gold lattice; scale bar 500 nm [95]. (**e**) Precise positioning of DNA origami triangles (127 nm edge) at predefined spots in photonic crystal cavity. Adapted with permission from [142]. Copyright 2016 Springer: Nature. (**f**) A schematic representation of precise positioning of DNA origami in small holes (diameter up to 200 nm) in thin metal films [143]. (**g**) Schematic representation of FIB patterning and covalent attachment of amino-terminated DNA triangles on the patterned surface via amidation reaction [144]. Adapted with permission from [138] copyright 2018, [95] copyright 2010, [143] copyright 2014 American Chemical Society.

The simplest and most straightforward approach is to exploit a negatively charged surface and use divalent cations, such as Mg^2+^ or Ni^2+^, to bridge the negative DNA structure and surface, as discussed above and shown in Figure 2c. However, most of the technologically attractive surfaces, including Si/SiO_2_, graphene, Teflon, or gold, require additional surface functionalization to render the charge for better adsorption and, in particular, to enable patterning. Kershner et al. [132] combined electron beam lithography and oxygen etching in order to create binding sites for triangular DNA origami, as presented in Figure 7a. A SiO_2_ surface was rendered hydrophobic by functionalizing it with a monolayer of hydrophobic trimethylsilyl (TMS) or with a thicker layer of diamond-like carbon (DLC). The patterned areas exposed to O_2_ plasma or UV-ozone became hydrophilic and exhibited high selectivity for attaching DNA origami by Mg^2+^-mediated binding. Later, they improved the method and demonstrated the importance of using ethanol in the adsorption of DNA structures. Rinsing the sample only with water led to the desorption of DNA from the surfaces, as for rinsing with ethanol/water mixture led to the successful attachment of the origami [133,134].

Scientists have also demonstrated alternative ways to immobilize DNA origami without using high magnesium nor sodium concentration, which are not desired for electronic devices [135,136]. In both studies, the Si/SiO_2_ substrate was modified with aminosilanes, which resulted in a positively charged surface enabling the electrostatic immobilization of origami. Gerdon et al. [137] demonstrated a similar strategy in attaching DNA origami on lithographically fabricated gold islands. Flat gold islands on the Si wafer were functionalized with carboxylate-modified alkanethiol molecules through the Au-S bound and the other end having a carboxylic acid group was exploited to immobilize DNA structure via Mg^2+^-mediated binding.

Apart from the electron-beam lithography, there are other approaches to create periodic structures. For example, the group of Adrian Keller fabricated nanoholes in gold films using nanosphere lithography, which further enabled the adsorption of single DNA origami triangles on the underlying Si wafers again through the Mg^2+^ bridges (Figure 7b) [138]. Yun et al. [139] presented attachment of rectangular DNA origami on various types of patterned graphene oxide surfaces, in the presence of divalent Mg^2+^ bridging the structure and surface. Hydrophobic interactions have also been used to immobilize rectangular DNA origami on Teflon amorphous fluoropolymer (Teflon AF) [140]. In the study, porphyrin was conjugated to the origami structure to mediate the binding to hydrophobic Teflon forming square lattice with a minimum feature size of 40 nm and 1 µm pitch (Figure 7c).

An appealing strategy for selective alignment of immobilized DNA nanostructures to lithographically patterned surfaces is the use of covalent bonding. Covalent interactions offer plausible solutions to effectively guide the assembly process by chemically altering either the DNA structures or surfaces with functional groups. The most common DNA immobilization method is taking advantage of the wide possibilities offered by thiol-chemistry. Ding et al. [95] designed 6-helix DNA bundles, which were thiol-modified at both ends of the tube, and covalently bind them to the 60 nm size gold islands created by e-beam lithography. The arrangement of the islands led to the formation of several different types of gold periodic lattices bridged by DNA nanotubes; the hexagonal lattice as one of the examples is shown in Figure 7d.

Oligonucleotides have been also modified with amine and various hydrophobic molecules such as cholesterol, porphyrin, chromophores, and biotins. Gopinath et al. [135] devised the idea further and instead of electrostatic binding of DNA structures described above, the origami were modified with amine groups and attached to the carboxylate-terminated Si/SiO_2_ surface via amide bond formation. Recently, the same approach was taken up by Hawkes et al. [141] to organize DNA origami into nanoarrays and create a biomemetic surface. The lattice of rectangular SiO_2_ domains exposed by holes milled with focused ion beam on a metal film covering the substrate, were silanised with a carboxylic acid terminated silane. The amine groups, introduced to the origami formed an amide-bond, providing selective attachment.

Among others, fluorophores are widely used as markers to monitor dynamics or for visualization purposes. For instance, Gopinath et al. [142] used the above method to precisely position DNA origami featuring fluorescent dye (Cy5), at the desired positions in periodic photonic crystals (Figure 7e). Pibri et al. [143] also functionalized the DNA origami nanoadapter with a fluorescent dye to fit only a single dye into zero-mode waveguide (ZMWs), schematically presented in Figure 7f. They attached the biotin anchors to the other side of the origami nanoadapter to form a strong noncovalent binding with neutravidin proteins attached to the bottom glass of the ZMW. This set-up enabled probing molecular fluorescence and the effect of ZMW on it at the single-molecule level.

## 5. Future Prospective

### 5.1. Arranging Other Components by DNA Lattices

Since the first introduction of self-assembled DNA structures, a strong emphasis has been put on DNA lattice assemblies and further on utilizing them to organize other nano-objects or functional materials. Even the first goal of the founder of the DNA nanotechnology field, Ned Seeman, was to use the DNA self-assembly to form crystals out of molecules that do not crystallize otherwise [145]. This would allow him as a crystallographer to study those molecules. Later, along with the worldwide boom of plasmonics, the metallic nanoparticles naturally became the most attractive objects to arrange in higher-order lattices with the use of DNA [41,146]. The precise arrangement of the gold nanoparticles (AuNP) with the help of DNA was pioneered already in 1996 by Mirkin and Alivisatos, and coworkers [147,148], who functionalized AuNPs with thiolated DNA strands to hybridize them with complementary strands attached to other particles. This immediately resulted in the formation of dimers and trimers as well as bigger aggregates. Later the field has progressed in many different directions [149], and also many plasmonic constructions have been developed [146,150].

**Figure 8:**
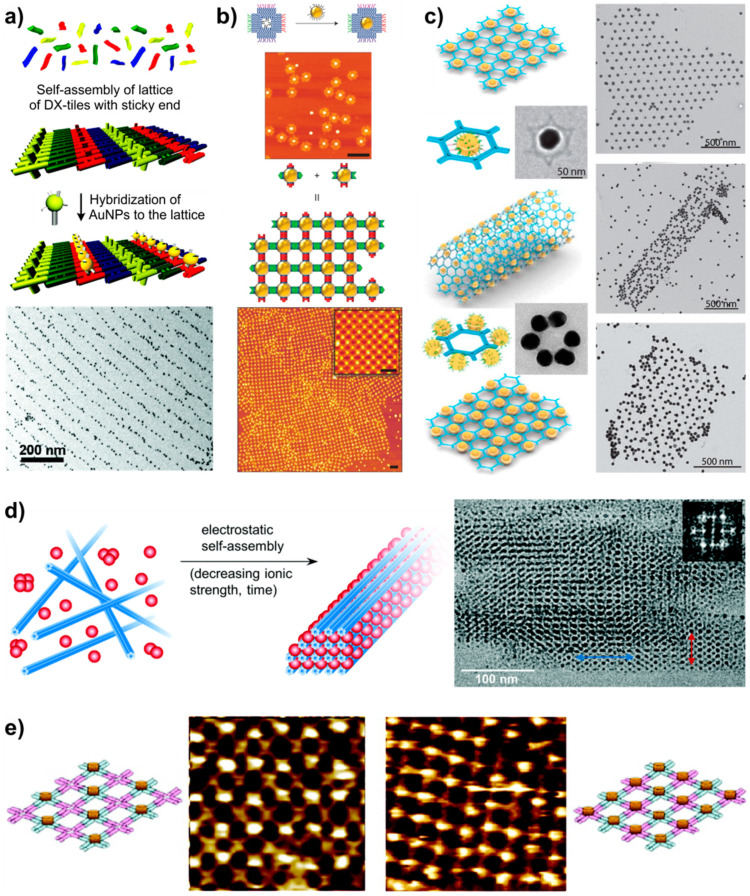
Using DNA-tile lattices to organize AuNPs and protein (**a**) DX-tile lattice for assembling AuNPs. The DX-tile lattice is first assembled in solution and immobilized on mica after which functionalized AuNPs are hybridized to a sticky end on the lattice. TEM image of the ready AuNP array [152]. (**b**) DNA origami framed AuNPs. Each AuNP is framed by DNA origami capable of attaching to other framed particles enabling lattice formation. AFM image of separate framed AuNPs as well as ready formed AuNP lattice. Scale bars are 200 nm. Adapted with permission from [111]. Copyright 2016 Springer: Nature Chemistry. (**c**) Hexagonal origami structures with a single AuNP attached in the middle or with six AuNPs symmetrically attached outside the main hexagon. 3D illustrations show the formed lattices with or without a twist. TEM images of single tiles and the readily formed structures [61]. (**d**) Electrostatic self-assembly of DNA origami and AuNPs. Crystal structures were formed upon dialysis against decreasing ionic strength. Cryo-TEM image of the formed crystal. Adapted with permission from [93] The Royal Society of Chemistry. (**e**) Precise control of periodic spacing between individual proteins by programming the self-assembly of cross-shaped DNA tiles [154]. Adapted with permission from [152] copyright 2004, [61] copyright 2016, [154] copyright 2005 American Chemical Society.

The first arrangement of AuNPs with the help of self-assembled DX-tile lattice was produced by Kiehl et al. [151] in 2002. They utilized the original DX-tile lattice design [48,67] with some of the tiles modified with an additional sticky end as an attachment site for ssDNA functionalized 1.4 nm diameter gold nanoparticles. The DX-tile lattice was first assembled on a mica substrate following the procedures discussed above, and after that, the functionalized AuNPs were precisely positioned via hybridization of complementary strands. Later the same group extended the methods to selectively arrange different sizes of AuNP to chosen locations [152,153], as shown in Figure 8a. Similar ideas but with different DNA motifs were utilized by Yan et al. [100], who utilized their previously developed lattice from cross-shaped tiles [79] but now with an additional sticky end modification, and Seeman et al. [81] who used 3D-DX triangle motifs to organize two different sized AuNPs on a periodic lattice. The former AuNP decorated lattice (the cross-shaped tiles) was prepared in two steps like in the work of Kiehl above. But in the latter case, the DNA-motif-AuNP hybrid was prepared in one step, where the 3D-DX triangles and the AuNP hybridization happened at the same time. The assembled triangles with only a single AuNP attached were gel-purified and isolated. The collected 3D-DX triangle-AuNP conjugates were then mixed with the complementary 3D-DX triangles, to form a two-triangle array. Also, Mao and coworkers utilized a self-assembly process of 4-point star motifs into a 2D lattice, which was glued to mica by Ni^2+^ ions. After that, the cavities were used to attach AuNPs as shown above in Figure 5c [115].

Liu et al. [111] demonstrated a clever strategy to organize particles of different sizes by designing a DNA frame from cross-shaped DNA origami tile components, as illustrated in Figure 8b. The precise arrangement of nanoparticles enables the formation of diverse architectures, both finite and infinite, and the manipulation of selective interactions. Similarly, both finite and infinite honeycomb lattices with AuNPs were formed by special hexagonal wireframe origami [61]. In their work Ke et al. formed two types of hexagonal origami structures; one with a single-AuNP attached in the center and another with six AuNPs symmetrically attached outside the main hexagon. Structures are shown in Figure 8c together with the lattices formed out of them by the joints between the protruding DNA helixes at each corner of the hexagon. In addition, they fabricated twisted structures, which formed 3D tubes during the lattice formation (Figure 8c). Twist was induced to the structure by intentionally skipping some bases in the design of the DNA origami staple strands. 3D AuNP tubes were also formed by Yan et al. [155] but using regular DX-tiles. Even more complex wireframe origami-based 3D DNA-AuNP-structures were formed by Gang and coworkers [156,157]. On the other hand, Kostiainen et al. [93] formed 3D structures out of functionalized AuNPs and six-helix DNA bundles only based on electrostatic interactions as shown in Figure 8d.

In addition to gold nanoparticles, DNA tile lattices have been used for the periodic arrangement of proteins. In their early work with the cross-shaped tiles, LaBean et al. [79] attached streptavidin to each crossing of the formed lattice. This was one of the first DNA assembled protein arrays. A few years later the Yan extended the method to selectively attach the proteins to various tiles forming designed protein patterns [154] as shown in Figure 8e. Later the Keller group utilized their close-packed lattice from Rothemund triangles to selectively attach Redβ proteins to the open surfaces at the lattice cavities [116] as shown above in Figure 5d. A different kind of approach was used by Chaput and coworkers who fabricated DNA self-assembled peptide nanoarrays for studying protein-protein interactions [158]. Park et al. [159] have also formed more complicated structures, which resemble salt crystal, out of AuNPs and Qβ-phage capsid particles by using DNA self-assembly to guide the assembly.

### 5.2. Outlook

Structural DNA nanotechnology has been crossing the boundaries of biology, chemistry, physics, and engineering for over 30 years. This intersection of the fields is the essence of the modern nanotechnology, which is providing potential for numerous new applications, especially within molecular scale phenomena and devices. For the progress of the field, DNA has been one of the most intriguing components. The self-assembly methods provided by the DNA nanotechnology have made it significantly easier to construct nanoscale objects with unparalleled precision and programmability giving rise to unique approaches and solutions to challenges in science and technology. This achievement may be the key to create superior functional materials [160], e.g., for biotechnology [161], electronics [162], as well as optics and photonics [146,150]. Even the DNA computation is still mostly in its infancy, future developments may yield it a more comprehensive role in bio-computation and data storage [163]. In addition, as a biomaterial, DNA assemblies have enormous possibilities for drug delivery, which has become one of the leading fields of DNA nanotechnology [22,164].

Especially alluring are the novel techniques combining DNA self-assembly with other modern nanofabrication techniques. It has already been shown that DNA-based nanostructure can serve as a great template, e.g., for nanolithography [44], or for fabrication of semiconducting electrical devices [165]. It is therefore essential for science and technology to continuously keep improving the development of DNA nanotechnology and explore its possibilities beyond the applications so far. In this progress, the DNA-tile lattices have a key role as they provide the highest possibility to build larger and more complex structures to bridge the gap between the molecular assemblies and regular technologies and engineering.

## Data Availability

Not applicable.
